# Neutrophil NLRP3 promotes cardiac injury following acute myocardial infarction through IL-1β production, VWF release and NET deposition in the myocardium

**DOI:** 10.1038/s41598-024-64710-4

**Published:** 2024-06-24

**Authors:** Lukas A. Heger, Nicolas Schommer, Stijn Van Bruggen, Casey E. Sheehy, William Chan, Denisa D. Wagner

**Affiliations:** 1 Program in Cellular and Molecular Medicine, Boston Children’s Hospital, 1 Blackfan Circle, Ninth Floor, Boston, MA 02115 USA; 2 Department of Pediatrics, Harvard Medical School, Boston, MA 02115 USA; 3 Departement of Cardiology and Angiology, University Hospital Freiburg Bad Krozingen, 79106 Freiburg, Germany; 4 Center of Molecular and Vascular Biology, Department of Cardiovascular Science, KU Leuven, 3000 Leuven, Belgium; 5 Division of Hematology/Oncology, Boston Children’s Hospital, Boston, MA 02115 USA

**Keywords:** Molecular medicine, Risk factors

## Abstract

NLRP3 inflammasome has been implicated in neutrophil polarization and extrusion of neutrophil extracellular traps (NETs) in vitro and facilitates secretion of Il1-beta (IL-1β). Permanent ligation of the left anterior descending artery was used to induce MI in WT and NLRP3^−/−^ mice as well as in NLRP3^−/−^ recipient mice transfused with either WT or NLRP3^−/−^ neutrophils. NLRP3 deficiency reduced infarct size to roughly a third of WT heart injury and preserved left ventricular (LV) function at 12 h after MI as assessed by echocardiography and triphenyltetrazolium chloride staining of live tissue. Transfusion of WT but not NLRP3^−/−^ neutrophils after MI increased infarct size in NLRP3^−/−^ mice and significantly reduced LV function. The key features of myocardial tissue in WT neutrophil transfused recipients were increased H3Cit-positive deposits with NET-like morphology and increased tissue levels of IL-1β and plasma levels of von Willebrand Factor (VWF). Flow cytometry analysis also revealed that neutrophil NLRP3 increased the number of labeled and transfused neutrophils in the bone marrow of recipient mice following MI. Our data suggest a key role for neutrophil NLRP3 in the production of IL-1β and deposition of NETs in cardiac tissue exacerbating injury following MI. We provide evidence for a link between neutrophil NLRP3 and VWF release likely enhancing thromboinflammation in the heart. Neutrophil NLRP3 deficiency conferred similar cardioprotective effects to general NLRP3 deletion in MI rendering anti-neutrophil NLRP3 therapy a promising target for early cardioprotective treatment.

## Introduction

Acute myocardial infarction (MI) remains the leading cause of morbidity and mortality worldwide. The early inflammatory response following MI significantly influences the process of post-MI repair, and thus the severity of the resulting ischemic heart failure (HF) ^1^. Novel therapeutic targets are needed to minimize inflammation-mediated myocardial damage and improve clinical outcomes following MI.

The intracellular multi-protein complex assembled around NACHT, leucine-rich repeat (LRR), and pyrin domain (PYD)-containing protein 3—called NLRP3 inflammasome—is a critical component of the innate immune system. It is associated with the sterile inflammatory response that exacerbates post-MI injury ^1^. The NLRP3 inflammasome triggers the activation of caspase-1, activating the proinflammatory cytokines IL-1β and IL-18. Caspase-1 also cleaves gasdermin D (GSDMD), promoting the formation of membrane pores and pyroptosis. Chronic activation and gain-of-function mutations of NLRP3 have been associated with a broad spectrum of inflammatory disorders, including cardiovascular disease ^2^. Components of the NLRP3 inflammasome are upregulated in infiltrating leukocytes and in border zone cardiomyocytes shortly after MI ^3^. The inhibition of NLRP3 and other inflammasome components (apoptosis speck-like protein [ASC], caspase-1) or its cytokine end-products (IL-1β/IL-18) reduces infarct size in mice ^4–7^. The exact mechanisms and cell types conferring this cardioprotection, however, remain elusive.

Our lab has recently implicated the activation of neutrophil NLRP3 inflammasome in the release of neutrophil extracellular traps (NETs) in sterile inflammation. ^8^. Like the NLRP3 inflammasome, NETs released from activated neutrophils are a key driver of the early thromboinflammatory response following MI ^9–11^. NETs are decondensed chromatin structures decorated with cytotoxic proteins capable of exerting pro-inflammatory and pro-thrombotic activities in the extracellular space ^12^. NETs have been shown to prime macrophages and neutrophils to produce IL-1β in an NLRP3 inflammasome-dependent manner ^13,14^. Taken together, this suggests a vicious cycle, wherein the endothelial activator IL-1β promotes the synthesis of endothelial adhesion molecules, amplifies leukocyte recruitment and NET release, and thus further propels IL-1β production and thromboinflammation ^15,16^. Emerging experimental evidence is also linking neutrophil NLRP3 with MI-induced emergency granulopoiesis in the bone marrow (BM); murine studies have shown that MI promotes greater accumulation of activated neutrophils in BM, which secrete IL-1β and promote granulopoiesis. ^17,18^. This is particularly of interest since our lab just implicated NLRP3 inflammasome in neutrophil chemotaxis ^19^.

We therefore hypothesize that neutrophil NLRP3 plays a fundamental role in the detrimental early inflammatory response following MI through increased IL-1β production and NET release as well as in governing neutrophil recruitment.

## Results

### Global NLRP3 deficiency reduces infarct size and dampens emergency granulopoiesis following MI

**Figure 1:**
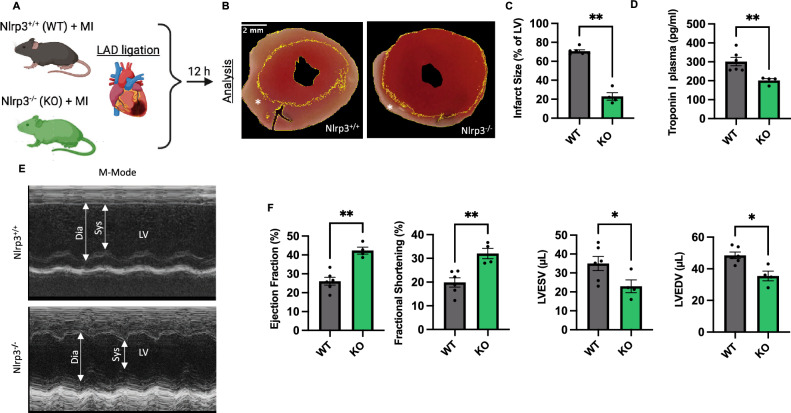
Nlrp3 deficiency alleviates infarct size in the early inflammatory phase. (**A**) Depiction of the experimental design: Ligation of the left anterior descending artery (LAD) in WT/NLRP3^+/+^ and KO/NLRP3^−/−^ was used to induce MI. Mice were evaluated for heart function and euthanized ≈ 12 h post-MI. (**B**) Representative pictures of Triphenyl tetrazolium chloride (TTC) staining at 12 h post MI with viable myocardium staining red and infarcted/necrotic area white (*) (scale bar 2 mm). (**C**) Quantification of Infarct size defined as percent of non-TTC stained Left ventricular (LV) area calculated in ImageJ. (**D**) Representative M-Mode images, depicting end-systolic (Sys) and end-diastolic (Dia) left ventricular diameters, acquired using echocardiography, at indicated timepoints in WT/NLRP3^+/+^ and NLRP3^−/−^ mice after MI. (**E**) Ejection fraction (EF), Fractional Shortening (FS), left ventricular end-diastolic volume (LVEDV), and end-systolic (LVESV) volume as assessed by echocardiography at 10 h following MI. (**F**) Plasma levels of Troponin-I as measured by ELISA in WT/NLRP3^+/+^ and NLRP3^−/−^ mice with MI. *Data are mean* ± SEM. *P < 0.05, **P < 0.01, ***P < *0.001; Mann–Whitney U-test*

Wild type (NLRP3^+/+^) and NLRP3^−/−^ mice were subjected to permanent ligation of the left anterior descending (LAD) coronary artery and euthanized after 12 h (Fig. 1A). Quantitative analysis of TTC staining (Fig. 1B) showed that infarct size was three-fold larger in NLRP3^+/+^ than in NLRP3^−/−^ mice (70.7 ± 1.5% vs. 22.92 ± 3.9%; n = 6 vs. 4; *p* < 0,01) (Fig. 1C). Similar results have been described for pharmacologic inhibition of the NLRP3 inflammasome in a non-reperfused myocardial infarction model after 7 days ^20^. Corresponding with the increased myocyte cell death and increased infarct size, circulating troponin I levels in plasma were elevated in NLRP3^+/+^ when compared to NLRP3^−/−^ mice (301.0 ± 21.9 pg/ml vs. 201.4 ± 9.8 pg/ml; n = 4 vs. 6; *p* = 0.009) (Fig. 1D). In line with the reduced infarct size, the NLRP3^−/−^ hearts had a preserved left ventricular (LV) systolic function post-MI, as determined by echocardiography, with better LV ejection fraction (42.3 ± 1.9% vs. 26.1 ± 2.1%; *p* = 0.009) and fractional shortening (FS) than the NLRP3^+/+^ hearts (32.1 ± 2.2% vs. 19.9 ± 2.0%; *p* = 0.009). Additionally, NLRP3^-/-^ animals exhibited decreased left ventricular (LV) end-diastolic volume (LVEDV) (35.5 ± 3.1% vs. 48.5 ± 2.1; *p* = 0.02) and end-systolic volume (LVESV) (22.9 ± 3.4% vs. 35.0 ± 3.7%; n = 6 vs. 4; *p* = 0.03) when compared to NLRP3^+/+^ mice, indicative of reduced adverse postinfarction remodeling and reduced LV dilation (Fig. 1E, F).

There is no difference in immune cell composition at baseline between NLRP3^−/−^ and WT ^21^. When looking at neutrophil homeostasis and emergency granulopoiesis following MI, we found a slight reduction in the number of bone marrow neutrophils when comparing NLRP3^−/−^ and NLRP3^+/+^ mice defined as Ly6G^+^ cells out of myeloid cells (CD45^+^CD11b^+^) in percent (62.6 ± 0.7% vs. 68.9 ± 0.5%; n = 4 vs. 6; *p* < 0.0001) indicative of a reduced emergency granulopoiesis in NLRP3^−/−^ mice following MI. At the same time, percentages of myeloid (CD11b^+^ of CD45^+^ cells in %) cells in the BM were not significantly different between genotypes (55.5 ± 4.3% vs. 47.5 ± 1.5%; n = 4 vs. 6; *p* = 0.1) (Supplement Fig. 1A,B). This is in contrast to our results showing increased platelet levels (770.5 ± 4.2 vs. 603.2 ± 54.0; n = 4 vs. 6; *p* < 0,01) with increased mean platelet volumes (MPV) (5.1 ± 0.1 vs. 4.8 ± 0.1; n = 4 vs. 6; *p* < 0,05) as well as slightly elevated hemoglobin levels (11.2 ± 0.5 vs. 9.8 ± 0.3; n = 4 vs. 6; *p* < 0,05) in NLRP3^-/-^ mice compared to WT following MI (Supplement Fig. 2A, 3A) ^22^. In summary, global NLRP3 reduced infarct size and improved post-infarction outcomes.

**Figure 2:**
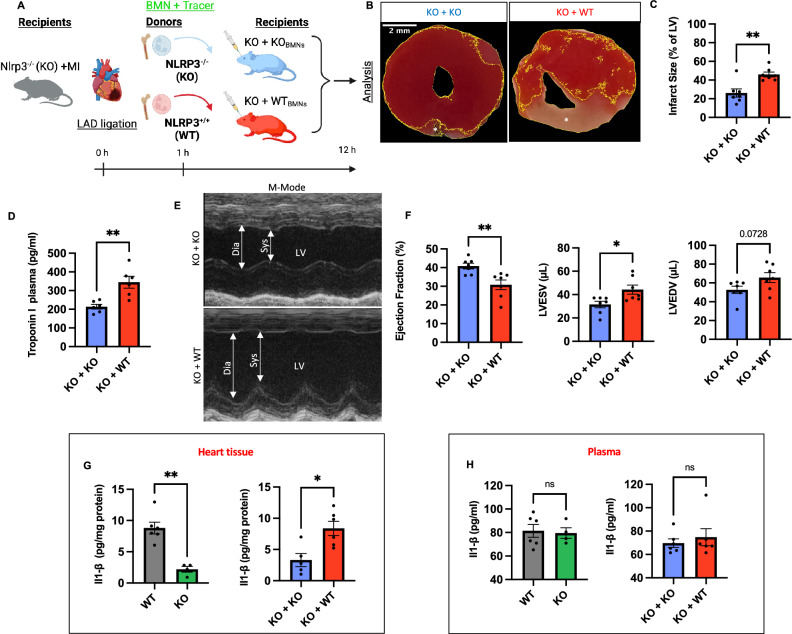
NLRP3^+/+^ neutrophils restore adverse infarct outcome in Nlrp3^−/−^ mice and provide a major source of IL-1β in infarcted myocardium. (**A**) Experimental design depicting the strategy for transfer of neutrophils isolated from WT and NLRP3^−/−^ (KO) mice (donors) into NLRP3^−/−^ mice (recipients) (n = 7). Purified neutrophils (≈ 99.4%) from the bone marrow (BM) of donors were pooled, labeled with CellTrace Carboxyfluorescein succinimidyl ester (CFSE), and injected in equal numbers into NLRP3^−/−^ recipients then subjected to MI (LAD-Ligation). Blood from the recipients was collected and echocardiographic analysis was performed at termination (≈ 12 h post-MI). (**B**) Representative pictures of TTC staining at 12 h post MI with viable myocardium staining red and infarcted/necrotic area staining white (*) (scale bar 2 mm). (**C**) Comparative analysis of infarct size in both groups defined as percent of non-TTC stained LV area calculated in ImageJ. (**D**) Quantitative comparison of plasma levels of Troponin-I determined using ELISA. (**E**) Representative M-Mode images, acquired using echocardiography. Systole (Sys), Diastole (Dia) and LV are marked with arrows. (**F**) Ejection fraction (EF), left ventricular end-diastolic (LVEDV) and end-systolic (LVESV) volume as assessed by echocardiography. (**G**) Quantitative comparison of heart tissue levels of IL-1β determined using ELISA. (**H**) Quantitative comparison of plasma levels of IL-1β determined using ELISA. *Data are mean* ± SEM. *P < 0.05, **P < 0.01, ***P < *0.001; Mann–Whitney U-test.*

### Neutrophil NLRP3 function is decisive for infarct severity and cardiac IL-1β production in the inflammatory phase following MI

To gain a deeper understanding of the potential deleterious impact specific to the neutrophil NLRP3 on the severity of myocardial damage, we conducted a neutrophil transfusion experiment. In this, we infused NLRP3^−/−^ mice with either WT or NLRP3^−/−^ bone marrow-derived neutrophils as outlined in the depiction of the experimental design (Fig. 2A). Recipient NLRP3^-/-^ animals were transfused with donor neutrophils to a level equivalent to the normal blood neutrophil counts (~ 2.5 × 10^6^ cells ml^−1^) one hour after the ligation of the LAD inducing MI.

We observed that transfusion of NLRP3^+/+^ neutrophils, but not NLRP3^−/−^ neutrophils, led to a significant increase in infarct size in the NLRP3^−/−^ mice determined via TTC staining as % of LV (46.0 ± 2.6% vs. 26.1 ± 4.4%; n = 7; *p* < 0.01) (Fig. 2B, C). The resulting infarct size in recipients transfused with NLRP3^+/+^ neutrophils was not significantly different from that observed in non-transfused NLRP3^+/+^ mice (70.7 ± 1.5%; n = 6 vs. 7; *p* = 0.4). Additionally, transfusion of NLRP3^+/+^ neutrophils also markedly increased post-MI Troponin I levels when compared to NLRP3^-/-^-neutrophil-transfused mice, comparable with levels measured in WT mice with MI (344.7 ± 31.4 pg/ml vs. 213.5 ± 12.7 pg/ml; *p* = 0.004; n = 6–7) (Fig. 2D). Correspondingly, NLRP3^+/+^-neutrophil-transfused mice demonstrated significantly reduced LV functions at 12 h after MI when compared to NLRP3^−/−^-neutrophil-transfused mice (30.8 ± 2.6% vs. 40.8 ± 1.5% EF; n = 7; *p* = 0.006) (Fig. 2E, F). Taken together, these data suggest that neutrophil NLRP3 plays a pivotal role for the detrimental effects of the early inflammatory phase following MI.

Considering the prominent role of the NLRP3 inflammasome in IL-1β production in general, we postulated the neutrophil NLRP3 inflammasome would serve as a major source of IL-1β in the early infarcted myocardium since neutrophils are not only the most abundant white blood cell type but also the first responders at the site of injury ^23–25^. To investigate this, we quantified IL-1β levels in heart tissue lysates and plasma samples after MI using ELISA (Fig. 2G, H). While plasma levels were similar (Fig. 2H), our results show a significant increase in IL-1β levels in heart tissues of NLRP3^+/+^ mice when compared to NLRP3^−/−^ mice (8.8 ± 0.9 pg/mg vs. 2.2 ± 0.5 pg/mg; n = 6 vs. 4; *p* = 0.009). Likewise, NLRP3^+/+^-transfused NLRP3^-/-^ mice exhibited higher levels of IL-1β in heart tissue in comparison to NLRP3^-/-^ mice transfused with NLRP3^-/-^ neutrophils (8.4 ± 1.1 pg/mg vs. 3.3 ± 1.0 pg/mg; n = 6 vs. 5; *p* < 0.5). To summarize, not only were we able to reconstitute infarct size by transfusion of NLRP3^+/+^ neutrophils into NLRP3^−/−^ animals but also elevate cardiac IL-1β levels to a degree similar to that observed in infarcted WT mice.

### Neutrophil NLRP3 promotes the release of NETs in myocardium and is involved in the migration of neutrophils to the myocardium and bone marrow post-MI

**Figure 3:**
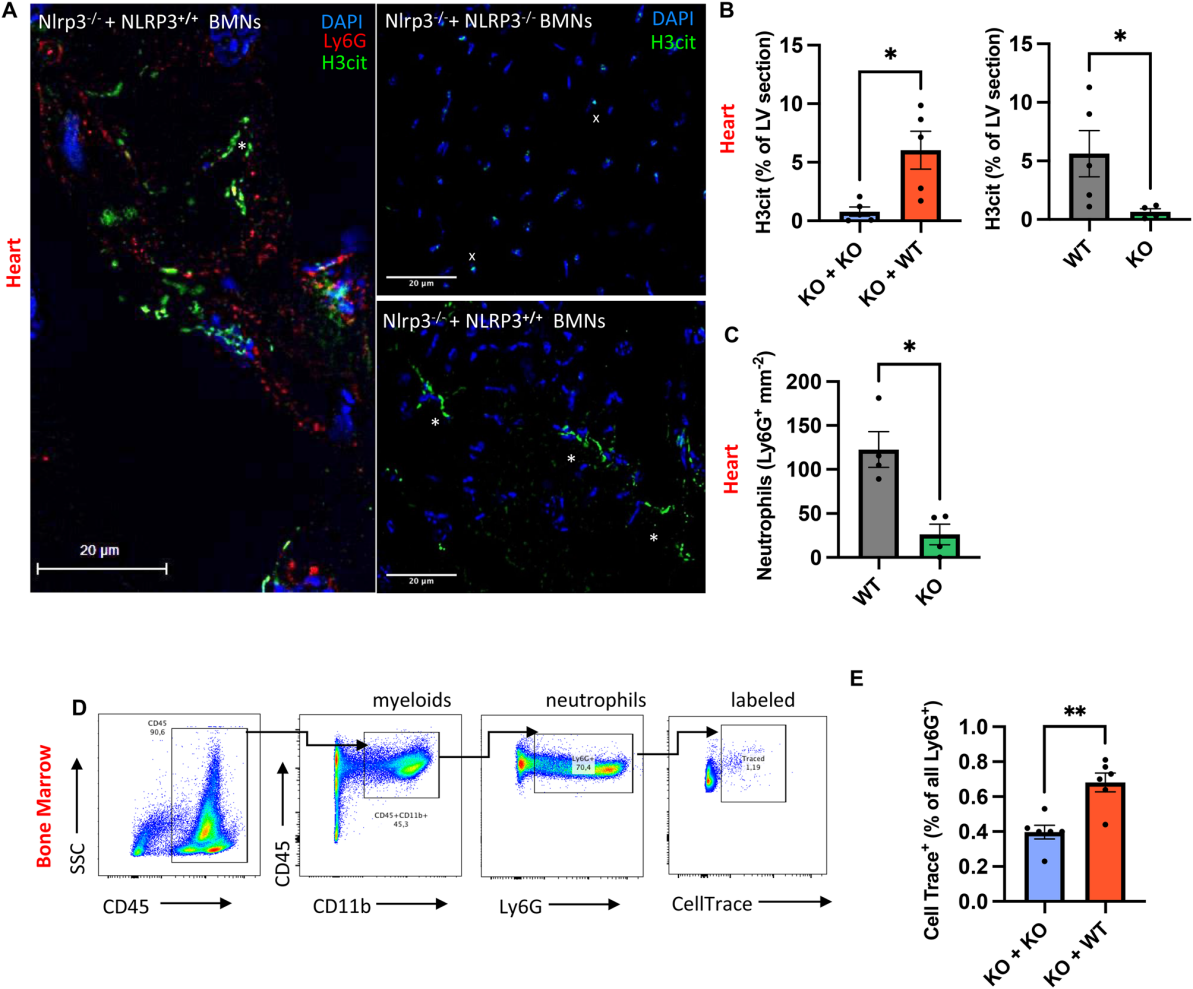
Neutrophil NLRP3 promotes NETosis in ischemic myocardium and homing to the BM in the early inflammatory phase. (**A**) Representative image of LV section of recipients transfused with NLRP3^+/+^ donor neutrophils immunostained for DAPI (blue), H3Cit (green) and Ly6G (red) at 100X magnification (left). Representative images at 50X magnification of LV sections of recipients transfused with either NLRP3^−/−^ (top right) or NLRP3^+/+^ neutrophils (down right) immunostained for DAPI (blue) and H3Cit (green). Extracellular H3Cit (*) next to Ly6G^+^ cells are considered NETs. Intranuclear staining of H3Cit (^x^) is considered citrullination of Histone 3 without NET release (scale bar 20 µm). (**B**) Comparative analysis of percent of total extracellular H3Cit^+^ (green) in LV heart sections quantified by ImageJ. (**C**) Comparative analysis of total Ly6G^+^ cell counts (per mm^2^) of LV heart sections counted manually by two independent operators in ImageJ. (**D**) Flow cytometry gating strategy used for isolated bone marrow cells. CD11b^+^ and CD45^+^ double-positive events were considered myeloid cells and Ly6G^+^ (out of CD11b^+^ and CD45^+^/myeloid cells) were considered neutrophils. Neutrophils (Ly6G^+^CD11b^+^CD45^+^) staining positive for CellTrace CFSE were considered originally labeled and transfused bone marrow neutrophils (BMN). (**E**) Quantitative comparison of originally labeled and transfused bone marrow neutrophils (% of CellTrace^+^ events out of Ly6G^+^ [CD11b^+^CD45^+^] events). *Data are mean* ± SEM. *P < 0.05, **P < 0.01, ***P < *0.001; Mann–Whitney U-test.*

Our lab has previously demonstrated a link between the neutrophil NLRP3 inflammasome and the release of NETs after neutrophil activation through in vitro studies ^8^. Drawing from this, we hypothesized that NLRP3 deficiency would result in decreased NET formation in injured heart tissue and additionally, that this would rely on neutrophil intrinsic NLRP3. Indeed, immunofluorescence staining of heart sections from NLRP3^-/-^ mice compared to WT mice showed significantly decreased amounts of citrullinated Histone 3 (H3Cit^+^) in vicinity to Ly6G^+^ cells in cardiac tissue, indicative of decreased amounts of NETs (5.6 ± 2.0% vs. 0.65 ± 0.27% extracellular H3Cit of LV; n = 5 vs. 4; *p* = 0.03) (Fig. 3A, B). We were able to confirm this in our transfusion experiment with increased deposition of NETs in NLRP3^−/−^ mice transfused with NLRP3^+/+^ neutrophils compared to mice transfused with NLRP3^−/−^ neutrophils (6.03 ± 1.6% vs. 0.79 ± 0.39%, n = 5; *p* = 0.02) (Fig. 3A, B). Of note, there was no difference in extracellular H3Cit deposition between WT and NLRP3^−/−^ mice transfused with NLRP3^+/+^ neutrophils and between NLRP3^−/−^ mice and NLRP3^−/−^ mice transfused with NLRP3^-/-^ neutrophils. Thus, the increase in extracellular H3Cit deposition was due to the effect of neutrophil-derived NLRP3. H3Cit is predominantly deposited extracellularly in the presence of NLRP3 in neutrophils. However, in the absence of NLRP3 in neutrophils, there is almost no staining outside the nucleus and only weak staining in the nuclei in a few segments of the nuclear chromatin (DAPI^+^) (Fig. 3A). Tissue hypoxia and necrosis impair the vascular endothelial cell integrity and augment massive recruitment and infiltration of neutrophils early after MI. There is new strong evidence implicating NLRP3 inflammasome in chemotaxis of neutrophils ^19^. While there was no significant difference in infiltrating neutrophils in whole hearts post-MI measured by FC (Fig. 3D, E), IF staining of LV sections of infarcted hearts for the neutrophil marker Ly6G showed a significant reduction of neutrophils in NLRP3^−/−^ mice compared to WT (26.2 ± 11.8 vs. 122.6 ± 20.3; n = 4; *p* < 0.05) supporting a role for NLRP3 in neutrophil recruitment to the infarcted heart (Fig. 3C). There was no difference in peripheral immune cell counts between both groups (Supplement Fig. 2B, 3B). To further evaluate whether NLRP3 deficiency would affect homing of transfused neutrophils, isolated neutrophils were labeled (with CellTrace Carboxyfluorescein succinimidyl ester [CFSE]) ex vivo before transfusion (Fig. 2A, refer to methods). We found most labeled neutrophils in the bone marrow, particularly with significantly higher numbers in recipient animals transfused with NLRP3^+/+^ neutrophils (0.68 ± 0,0539% vs. 0.40 ± 0,0392%; n = 6; *p* < 0.01) (Fig. 3D). This is in line with previous reports implicating neutrophil NLRP3 inflammasome in chemotaxis and reverse-migration to the BM with subsequent induction of granulopoiesis ^18,19^. Interestingly, we could not find any CFSE labeled transfused neutrophils in the peripheral blood and total numbers of CFSE labeled transfused neutrophils in heart tissue had a tendency to be lower in KO animals transfused with NLRP3^+/+^ neutrophils measured by FC (13.6% ± 1.5 vs. 27.6 ± 6.0, n = 4, *p* = 0.06) (Supplement Fig. 1C,D). This is in line with our data suggesting that NLRP3 promotes NETosis and facilitates neutrophil recruitment.

### Neutrophil NLRP3-Inflammasome exacerbates endothelial activation with increased VWF levels and ICAM-1 expression

**Figure 4:**
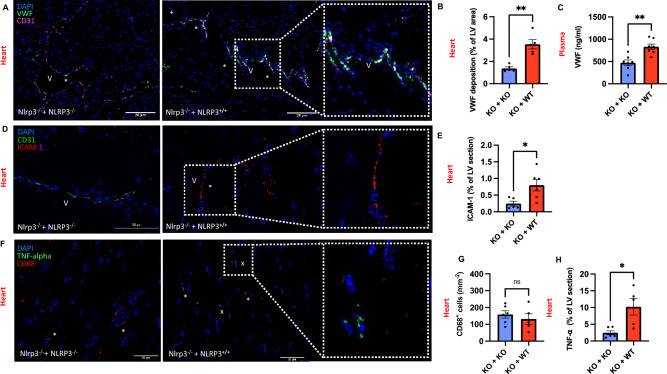
Neutrophil NLRP3 exacerbates endothelial activation with increased VWF deposition and ICAM-1 expression. (**A**) Representative images of LV sections of recipients transfused with either NLRP3^−/−^- or NLRP3^+/+^-neutrophils immunostained for DAPI (blue), von Willebrand Factor (VWF) (green), an endothelial adhesive protein deposited in the vasculature, and CD31 (pink), an endothelial cell marker (scale bar 20 µm). VWF deposits (*) in the Vessel lumen (V) (**B**) Comparative analysis of VWF deposition in % of LV sections quantified by ImageJ. (**C**) Quantitative comparison of plasma levels of VWF (ng/ml) determined using ELISA. (**D**) Representative images of LV sections of recipients transfused with either NLRP3^−/−^- or NLRP3^+/+^-neutrophils immunostained for DAPI (blue), CD31 (green) and ICAM-1 (intercellular adhesion molecule-1) (red), an endothelial adhesion molecule upregulated upon activation (scale bar 50 µm). Vessel lumen (V), ICAM-1 (*). (**E**) Comparative analysis of ICAM-1 and CD31 positive area in % of LV quantified by ImageJ. (**F**) Representative images of LV sections of recipients transfused with either NLRP3^−/−^- or NLRP3^+/+^-neutrophils immunostained for DAPI (blue), tumor necrosis factor α (TNF-α) (green)(^x^), a proinflammatory and cytotoxic mediator secreted by macrophages and CD68 (red) (*), a surface marker mainly expressed by monocytes and macrophages (scale bar 20 µm). (**G**) Comparative analysis of CD68^+^ cells relative to all cells (DAPI^+^) counted manually by two independent operators in ImageJ. (**H**) Comparative analysis of TNF-α positive area in % of LV quantified by ImageJ. *Data are mean* ± SEM. *P < 0.05, **P < 0.01, ***P < *0.001; Mann–Whitney U-test.*

As we observed elevated levels of both NETs and cardiac IL-1β in the presence of neutrophil NLRP3, we proceeded to investigate the potential downstream effects on endothelial activation. To assess recruitment and activation of monocytes/macrophages, we performed immunostaining for CD68, mainly expressed by monocytes and macrophages, and tumor necrosis factor α (TNF-α), a proinflammatory and cytotoxic mediator secreted by macrophages. TNF-α, as part of the early inflammatory phase, plays a significant role in the immune response following myocardial infarction ^26,27^. Its secretion contributes to the recruitment of immune cells and impacts cardiac tissue repair and remodeling. While we observed no significant difference in the number of CD68^+^ cells when comparing our transfused animals (131.2 ± 31.6 mm^-2^ vs. 158.3 ± 21.9; n = 5 vs. 6; *p* = 0.63), we found a significant increase in TNF-α levels in heart tissues of NLRP3^−/−^ mice transfused with NLRP3^+/+^ neutrophils compared with mice transfused with NLRP3^−/−^ neutrophils (10.23 ± 2.5% vs. 2.5 ± 0.6% of LV section; n = 5 vs. 6; *p* = 0.02) (Fig. 4F–H). This could partially be explained by the early time point of observation (12 h post-MI), during which the influx of neutrophils is peaking, but the major infiltration of monocytes is yet to come ^28^. We also found an upregulated ICAM-1 expression in heart tissues of NLRP3^−/−^ mice transfused with NLRP3^+/+^ neutrophils in comparison to mice transfused with NLRP3^-/-^ neutrophils (0.80 ± 0.17 vs. 0.25 ± 0.07; n = 6 vs. 5; *p* = 0.02) (Fig. 4D, E). Additionally, we performed IF stainings on LV sections to investigate the expression of endothelial markers including von-Willebrand Factor (VWF) (Fig. 4A–C) and ICAM-1/CD54 (Fig. 4D). These molecules play a critical role in leukocyte recruitment and transendothelial migration within the tissue. Interestingly, we detected a two-fold increase in VWF deposition in myocardial tissue in NLRP3^−/−^ mice transfused with NLRP3^+/+^ neutrophils when compared to mice transfused with NLRP3^−/−^ neutrophils (3.6 ± 0.4% vs. 1.4 ± 0.1% of LV tissue; n = 5; *p* = 0.008) (Fig. 4B). This was paralleled by increased VWF levels in plasma of NLRP3^+/+^-neutrophil-transfused mice as measured by ELISA (830.4 ± 62.6 ng/ml vs. 472.4 ± 63.4 ng/ml), thereby establishing a new link between neutrophil NLRP3 inflammasome and VWF secretion (Fig. 4C).

## Discussion

The NLRP3 inflammasome assembly is emerging as a key pathway in orchestrating the immune response following MI ^3,7,29^. Targeting the NLRP3/interleukin-1β innate immunity in cardiovascular disease has gained substantial interest, yielding advancements in both preclinical and the first clinical trials alike ^7,29–32^. Notably, the pioneering CANTOS trial has showcased improved outcomes in atherosclerosis patients treated with Canakinumab—a human monoclonal antibody against IL-1β—thereby establishing a tangible proof of concept ^33^. However, the current inflammasome research vastly regards monocytes/macrophages as the primary players, leaving the impact of neutrophil inflammasome largely unexplored and likely underappreciated. The present study shows that the neutrophil NLRP3 inflammasome is of crucial importance for infarct size in the early inflammatory phase of myocardial ischemic injury. We identify at least three functionally relevant pathways depending on neutrophilic NLRP3 inflammasome assembly in MI: endothelial activation with increased VWF and ICAM1 expression, extrusion of NETs, and IL-1β production. Our data show an increased number of NETs deposited in the vicinity of the infarcted area of both WT and NLRP3^−/−^ mice transfused with NLRP3^+/+^ neutrophils. This is in line with our in-vitro study showing that neutrophil NLRP3 is a strong regulator of NETosis ^8^. In humans, key components of NETs have been found at the culprit lesion site of MI and correlate with infarct size ^34,35^. Due to their nature, NETs are decorated with a plurality of pro-thrombotic or pro-inflammatory molecules, proteases, and reactive oxygen species. They have been described to induce the differentiation of cardiac fibroblasts and recruit platelets, thus promoting pathogenic cardiac fibrosis ^36,37^. Beyond that, NETs are recognized to trap functional tissue factor, induce platelet activation, and boost thrombin generation ^11,35^. The lack of neutrophil NLRP3, and subsequently of NETs, at the site of MI could limit the prothrombogenic response and help preserve the cardiac microcirculation. This is echoed by the beneficial effect of DNase in mitigating injury following MI ^9,10^. Inhibition or deficiency of NLRP3 has been shown to increase ischemic tolerance by attenuating apoptosis ^38–40^. Neutrophils are the prominent cell type in early MI, and they are known to produce IL-1β. Thus, the neutrophil NLRP3 inflammasome is well positioned to govern the extent of IL-1β and NET release in the inflammatory phase of MI. Our lab has also provided first evidence that peptidyl arginine deiminase 4 (PAD4), an enzyme critically involved in the citrullination of histones and subsequent NET formation, promotes NLRP3 inflammasome formation ^8,11^. MI dramatically increases PAD4 expression and activity in the heart, possibly advancing NLRP3 function ^41^. Our results add to the understanding of NET pathophysiology after MI, independent of PAD4 function. The exact mechanisms by which PAD4 and NLRP3 inflammasome support one another in MI and remodeling need to be addressed in future projects.

Our in-vivo non-reperfusion experiments prove that neutrophil NLRP3 is vital for cardiac IL-1β release following MI and support the notion that neutrophils are the major source of IL-1β in the early inflammatory response. Although in vitro studies show that the total amount of IL-1β released by each individual neutrophil is relatively low compared to macrophages, the substantial recruitment of neutrophils to the site of inflammation may compensate for this limitation ^42^. Furthermore, neutrophils are capable of a more rapid inflammasome assembly when compared to macrophages without the need for prior priming ^8^. Additionally, neutrophils lack the desensitization exhibited by macrophages within a DAMP-rich environment ^43^. Interestingly, recent evidence shows that key constituents of the NLRP3 inflammasome, particularly the ASC speck, possess the capacity to function as “prionoids,” triggering the activation of monocytes/macrophages and other neutrophils upon ingestion ^44–46^. In a snowball effect, the transfused NLRP3^+/+^ neutrophils could expel ASC speck to be absorbed by NLRP3 deficient cells, and subsequently support IL-1β release from other cells ^45^. This downstream activation of surrounding monocytes/macrophages could also be the cause for the increased TNF-α expression we observed in recipient mice transfused with NLRP3^+/+^ neutrophils (Fig. 4F, H). Our results suggest an autocrine feed-forward loop, with an activating interplay between NETs and neutrophil NLRP3-dependent synthesis of IL-1β. Interestingly, there was no significant difference in plasma IL-1β levels among the experimental groups. This suggests that neutrophils locally activate the NLRP3 inflammasome and release IL-1β, rather than in a systemic fashion, which is in line with our findings confirming neutrophils as important cellular mediators^48^.

Once activated and released, IL-1β is a major proinflammatory cytokine driving the synthesis of endothelial adhesion molecules ^15^. Correspondingly, we found increased signals for ICAM-1 expression in heart tissue of mice transfused with NLRP3^+/+^ neutrophils (Fig. 4D, E). However, there is only negative data on a potentially direct effect of IL-1β on synthesis or release of VWF. Endothelial VWF supports leukocyte and NET adhesion to the vessel wall, amplifying the local thromboinflammatory process ^11^. In this study, transfusion of NLRP3 competent neutrophils resulted in an increase in VWF plasma levels and increased endothelial VWF deposition (Fig. 4A–C) in KO animals following MI. Neutrophil NLRP3 stimulation of VWF release is a novel finding, possibly affecting VWF-mediated leukocyte recruitment at an early time point post-MI. One likely mechanistic explanation is that NLRP3 positive neutrophils are able to produce NETs which occlude the penumbra cardiac microvasculature, thus increasing the region of ischemia. Ischemia is known to stimulate Weibel-Palade body release (VWF and P-selectin), elevating VWF and promoting more neutrophil recruitment and propagation of tissue injury ^47^. Whether the effect of neutrophil NLRP3 on VWF release and endothelial ICAM-1 expression (Fig. 4D, E) are triggered by platelets stimulated by NETs remains elusive.

We found decreased amounts of neutrophils within the infarcted area of NLRP3 ^-/-^ mice post MI. Together with our lab’s recent report showing that NLRP3 in neutrophils directs chemotaxis in vitro and to a wound ^19^, we may propose that this is also the case in MI. Notably, we also found decreased amounts of neutrophils in the BM of NLRP3^−/−^ mice together with signs of reduced granulopoesis. These observations were reversed by transfusion of NLRP3-competent neutrophils. This is in line with recent experimental evidence pointing towards an important role of NLRP3 in emergency granulopoiesis following MI ^17,18^. Decoding whether the chemotactic signal differs when comparing BM to myocardium could be key to understand differences in neutrophil diversity and plasticity following MI. Our results suggest a prominent role of neutrophil NLRP3 for neutrophil homing and could have broad implications for various diseases, including cancer therapy, and should be investigated further in more detail. Our findings support an emerging concept for neutrophils as key cells that help to fine tune innate and adaptive immune responses following injury.

Our study demonstrates that neutrophil NLRP3 serves as a primary regulator of cardiac NETosis, IL-1β release, and VWF release following MI. As a result, it presents a compelling and promising target to inhibit excessive early neutrophil derived inflammation in acute MI.

## Conclusion

In summary, we provide exciting evidence that neutrophil NLRP3 exacerbates myocardial infarction (MI) injury in the early phase through the local release of NETs and IL-1β. We pinpoint neutrophil NLRP3 as a primary source of IL-1β in early ischemic myocardium. Our data indicate that neutrophil NLRP3 is vital for recruitment of neutrophils to the cardiac tissue and BM following MI. Finally, we show that functional neutrophil NLRP3 promotes VWF secretion, implicating it in VWF-mediated leukocyte/platelet recruitment. Targeting neutrophil NLRP3 and the pathways that help its assembly may be a new therapeutic strategy to reduce ischemia-related cardiac damage.

## Limitations

Through the various crosstalk mechanisms between pathways that promote NETosis, IL-1β, and NLRP3 inflammasome assembly, it cannot be differentiated between effects of impaired NET release and mere absence of NLRP3 inflammasome assembly. Also, we cannot rule out an impact of an increased recruitment of immune cells in NLRP3^+/+^ transfused mice facilitating caspase-1 independent IL-1β processing or the impact of cellular interactions downstream of NLRP3.

### Methods

The data that support the findings of this study are available from the corresponding author upon reasonable request. Experimental protocols were approved by the Institutional Animal Care and Use Committee of Boston Children’s Hospital (Protocol number: 20–01-4096R) in accordance with the ARRIVE guidelines.

#### Animals

Male C57BL/6J mice purchased from Jackson Laboratory and NLRP3^−/−^ mice on a C57BL/6J background were housed at the local animal facility of Boston Children’s Hospital. All mice were kept specific pathogen free. NLRP3^−/−^ mice and Wild Type C57BL/6J (NLRP3^+/+^) mice were subjected to permanent left anterior descending (LAD) artery ligation surgery to induce myocardial infarction (MI) and sacrificed after deep anesthesia to effect with the inhalant isoflurane (as determined of lack of response to footpad squeeze) followed by cervical dislocation one day after MI. Further NLRP3^−/−^ mice were randomly allocated to a transfusion experiment after induction of MI and either transfused with WT (NLRP3^+/+^) or NLRP3^−/−^ neutrophils and sacrificed one day after. Donor animals were male C57BL/6J mice or NLRP3^−/−^ mice sacrificed at the same time as the LAD ligation surgeries of the recipient animals. All recurring events including animal scoring were done at similar times during the day. All groups were age and sex matched and were fed ad libitum with free access to water. All procedures conform with the NIH Guide for the Care and Use of Laboratory Animals. Experimental protocols were approved by the Institutional Animal Care and Use Committee of Boston Children’s Hospital (Protocol number: 20-01-4096R).

#### Echocardiography

B-Mode, M-mode, and Doppler echocardiography were performed in Wild type (WT) and NLRP3^−/−^ as well as NLRP3^−/−^ mice transfused with NLRP3^+/+^ or NLRP3^−/−^ neutrophils as previously described ^48^. Anesthesia was induced with 3% isoflurane and maintained at 1.5–2% for the duration of the procedure. Warmed echo gel was placed on the shaved chest. Body temperature was regulated through a heat pad and heart rate measured with an electrocardiogram, both were kept consistent between experimental groups (37° Celcius and 400–500 bpm). Echocardiography images were recorded using a Vevo- 3100 imaging system with a 25–55-MHz linear probe (MX550D; VisualSonics, Toronto, Canada). Percentage of ejection fraction was calculated in parasternal long-axis view (PSLAX). M-mode was measured at the papillary muscle level in the short-axis (SAX) view. Parameters measured in M-Mode/SAX include: Fractional shortening (FS), Left Ventricular Posterior Wall thickness (LV PWd) in diastole, and Left Ventricular Internal Diameter in diastole (LVIDd). From these measured values, other estimates were calculated by computerized algorithm of the echocardiograph such as Left Ventricular Mass (LV Mass = 0.8 × (1.04 × (((LVEDD + IVSd + PWd)3—LVEDD3))) + 0.6) or relative wall thickness (RWT = 2 × PWd/LVEDD). All measurements were obtained in triplicate and averaged.

#### LAD ligation surgery

C57BL/6J (WT, NLRP3^+/+^), NLRP3^−/−^, and NLRP3^−/−^ mice transfused with either isolated NLRP3^+/+^ or NLRP3^-/-^ neutrophils respectively (1 h after LAD ligation) were all subjected to permanent ligation of the left anterior descending artery (LAD) as previously described ^49^. All survival surgeries used isoflurane in oxygen as the anesthetic plus intraperitoneal injection of 100 mg/kg body weight (bw) ketamine + 5 mg/kg bw xylazine (diluted in 9% Saline solution) to initiate anesthesia. 3–4% isoflurane was used for intubation and 1.5–2% to maintain a surgical level of anesthesia (as determined by lack of response to footpad squeeze). Sufficient analgesia during the operation as well as postoperatively was ensured by subcutaneous (s.c.) injection of the opioid Ethiqua XR. In short, MI was induced by permanently occluding the left anterior descending artery (LAD). Therefore, mice were intubated with a 22 G intravenous catheter, placed on a heated pad, and connected to a mouse ventilator that administers 2% isoflurane supplemented with oxygen (flow rate 0.4 L/min, tidal volume 260 micron/stroke and ventilation rate 130 strokes/min). Chest inflation, breath rate, and depth of anesthesia were monitored closely, and an ECG lead connected to an ADInstruments multichannel recorder interfaced with a computer running Power lab4/30 with data acquisition software (Labchat 7) recorded from few 27G needle electrodes inserted subcutaneously in the front legs and hind legs. With a left lateral thoracic incision, the chest was opened and retracted for better visualization of the beating heart. A 7.0 silk suture was passed under the LAD and ligated to constrict the blood flow without damage to the tissue. Ischemia was visualized microscopically and confirmed in ECG. The sutures were kept in place and provided a landmark to find the exact location when processing the heart tissues later. After inducing ischemia, the chest wall was closed, an intravenous catheter was inserted, and 4 ml of air were aspirated using a 10 ml syringe to evacuate any residual pneumothorax. Finally, the skin was closed with a 4–0 prolene suture, isoflurane was turned off and the mouse extubated once the gag reflex was regained. Mice were observed in a safe environment until they ambulated normally and then returned to their cages.

#### Immunostaining

Mice were sacrificed by cervical dislocation under deep anesthesia with isoflurane (3–4% in oxygen) after verification of sufficient analgesia by a lack of visible response to a footpad squeeze. Euthanasia was confirmed with monitoring the mouse for respirations for at least one minute after cervical dislocation. Hearts were collected and residual blood cleared by retrograde perfusion with 5 ml ice cold PBS buffer. For cryosectioning, tissue samples were snap frozen in O.C.T (Tissue-Tek; product Code 4583). Cryosections of 8-µm were fixed in 4% paraformaldehyde, permeabilized with 0.1% Triton, blocked in 3% BSA, and incubated overnight at 4 °C with the following primary antibodies: Anti-Ly6G (clone 1A8; BioLegend); anti-H3Cit (Abcam; ab5103); anti-CD31 (BD Pharmingen; 553,370); anti-VWF (DakoCytomation; P0226); anti-CD68 (Abcam; ab53444); anti-ICAM-1 (BioLegend; 116,101); and anti-TNF-alpha (Abcam; ab6671). After washing, sections were stained with the respective Alexa Fluor–conjugated secondary antibodies (Alexa Fluor 488 donkey anti–rabbit [Invitrogen; Cat. Nr. A21206] IgG and Alexa Fluor 555 goat anti–rat [Invitrogen; Cat. Nr. A21434] IgG; Alexa Fluor 647 goat anti-rabbit [Invitrogen; Cat. Nr. A21244]) and counterstained with Hoechst 33,342 (1:10,000; Invitrogen; Catalog number: H3570).

#### Bone marrow isolation

Tibias and femurs were carefully isolated from both legs following euthanasia, and any surrounding soft tissues were removed to ensure clean specimens. To facilitate cell recovery, the tibias and femurs were cut off at both ends to remove the epiphysis. Next, the bones were rinsed with at least 10 ml of HBSS (Hank's Balanced Salt Solution) using a 22G needle. The rinsing process was performed over a 70 µm cell strainer to collect the cell suspension into 50 ml Falcon tubes. Gentle scraping and a syringe plunger were used to aid cell passage through the strainer. After collecting the cell suspension, the samples were centrifuged for 12 min at 500 g at room temperature. Following centrifugation, the supernatant was carefully discarded, and the pellet was resuspended in 3 ml of 1X PBS (Phosphate-Buffered Saline) to create a homogeneous cell suspension. Samples were then further used for neutrophil isolation and transfusion or flow cytometry, respectively.

#### Neutrophil isolation

Bone marrow neutrophils were isolated as previously described ^50^. Briefly, prepared and washed bone marrow cells (see above) were resuspended in 1X PBS and layered onto a discontinuous Percoll gradient (78%/69%/52% made isotonic with the addition of 10X PBS, Merck). Samples were centrifuged for 32 min at 1500*g* at room temperature, without breaks. After several washing steps, isolated neutrophils were resuspended in RPMI + 10 mM HEPES (Gibco™; Catalog number: 15630080) and counted in a Neubauer counting chamber for consecutive transfusion experiments.

#### Neutrophil labeling and transfusion

Isolated neutrophils were labeled prior to transfusion using a Cell Tracker dye (CellTrace™ CFSE, invitrogen; Cat. No. C34554) and then transfused. Briefly, the cells were carefully resuspended in 150 µl of Cell Tracker solution at a final concentration of 5 µM in prewarmed 1X PBS (Phosphate-Buffered Saline) at 37 °C. Once resuspended in the Cell Tracker dye solution, the cells were incubated for 10 min at 37 °C in the dark. After the incubation, the labeled cells were washed twice with ice-cold RPMI 1640 1X medium, supplemented with 10% FBS (Fetal Bovine Serum) and 1% Penicillin/Streptomycin. The washing step was performed by centrifuging the cells at 1600*g* for 5 min at 4 °C. For experiments involving cells from the same genotypes and origin, samples were pooled from 3–4 mice to ensure sufficient cell numbers (~ 3 × 10^6^ neutrophils). Cell concentration was determined using a Neubauer chamber and kept consistent across experiments. For subsequent transfusion the labeled cells were resuspended in prewarmed 1X PBS at 37 °C and then administered via intra-retroorbital injection. Detection of the CellTrace™ CFSE dye was enabled through flow cytometry.

#### Flow cytometry

After sacrifice, the heart was immediately perfused with 5 mL ice-cold 0.05% EDTA in PBS. The perfusion needle was inserted into the LV and the right atrium cut with a scissor. Paleness of the coronary arteries and cardiac veins was visually verified before cardiac excision. Apical sections, representative of LV tissue, were removed, then placed into ice-cold PBS and afterwards minced. Minced sections were incubated in a solution containing 1.5 mg/mL collagenase (Merck, Product Nr. C8051) for 45 min at 37 °C. After filtration through a 70 μm cell strainer, the single-cell suspension was centrifuged and the pellet resuspended in Dulbecco’s Modified Eagle Medium (DMEM, Thermo Fisher Scientific—US; Catalog Number 11965092) topped up with cell debris remover (Milteny Biotec; Order Number 130-109-398) and PBS followed by another centrifugation at 3000*g*. This separated the mononuclear cells from cellular debris. After centrifugation, both the upper- and interphase were discarded. Bone marrow cells were isolated as described above and then resuspended in flow cytometry buffer (0.1% BSA, 2 mM EDTA in PBS) at a 1:6 dilution, and red blood cell lysis was achieved using ACK lysing buffer (Gibco A1049201). Cells from hearts were also resuspended in flow cytometry buffer. The single cell suspensions were blocked with Fc block (anti-mouseCD16/CD32, 1:100 dilution, BioLegend; TrueStain Fcx; Cat. No. 101320), followed by washing and staining with Anti-CD45-Pacific Blue (BioLegend, 109,819), Anti-Ly6G-PE (eBiosciences, 12-5931-82), Anti-CD11b-APC (BioLegend, 101,224), and viability staining (invitrogen; Cat. No. 65-0864-14). Samples were run on a BD LSR Fortessa (BD Biosciences, San Jose CA) using FACS Diva software and analyzed on FlowJo software (Ashland, OR). Once the doublets (by FSC-H vs. FSC-A) and dead cells (live vs. dead) were excluded, labeled neutrophils were identified as CD45^+^ CD11b^+^ Ly6G^+^ and CellTrace™ CFSE^+^ cells. Neutrophil and myeloid percentages in hearts and bone marrows were quantified according to gating strategy displayed (Fig. 3D).

#### Peripheral blood and plasma analysis

Blood was collected from anesthetized mice via the retroorbital sinus into EDTA-coated capillary tubes and analyzed by a Hemavet 950FS (Drew Scientific) for complete blood counts. Platelet-poor plasma was prepared immediately after blood collection by centrifuging anticoagulated whole blood for 5 min at 2300*g*. Plasma supernatant was carefully removed and centrifuged again for 10 min at 16,100*g* to remove any remaining blood cells. Plasma samples were immediately stored at − 80 °C until analysis.

#### Tissue lysate preparation

For tissue lysate, tissue was dissected, washed with PBS, and homogenized vigorously. Tissue was then added to RIPA Lysis and Extraction Buffer (Thermo Scientific™; Catalog number: 89900) and incubated for 30 min at 37 °C. Afterwards the tissue suspension was sonicated for 2–5 min in rounds of 10 s at time at a power of 180 watts. Sample was kept on ice throughout the whole process. Protein levels were determined using Bradford Assay Dye-based protein detection (Thermofisher Scientific; Catalogue Number 23236) according to manufacturer protocol.

#### Determination of plasma/tissue protein levels

Enzyme-linked immunosorbent assay (ELISA) was performed for Interleukin-1β (ELISA MAX™ Deluxe Set Mouse IL-1β; Catalogue Number 432616; BioLegend), Troponin I (CTNI elisa kit :: Mouse CTNI (Cardiac Troponin-I) ELISA Kit; Catalogue Number MBS766175; MyBioSource) and VWF (Mouse Von Willebrand Factor (vWF) ELISA Kit; Catalogue Number MBS2022796; MyBioSource) to determine protein plasma or tissue lysate levels.

#### Triphenytetrazoliumchloride staining

To measure infarct size, 2,3,5-triphenyltetrazolium chloride (TTC) staining was performed as described previously ^9^. After freezing the hearts at − 20 °C for 5 min, they were sectioned into 2 mm slices using a cold razor blade. These slices were then immersed in a phosphate buffer (0.2 mol/L Na2HPO4/0.2 mol/L NaH2PO4, adjusted to pH 7.4) containing 1% 2,3,5-triphenyltetrazolium chloride (Sigma-Aldrich Chemie, Steinheim, Germany). The sections were incubated in a water bath set to 37 °C for 15 min. During this incubation, necrotic tissue appeared white, while healthy myocardium was stained red. Following the staining process, the heart sections were carefully placed on a cover slip and embedded with 5% agarose. A second slip was then applied to cover the specimen securely. The slices were photographed from both sides and infarct size was determined using ImageJ software. The calculation involved measuring the percentage of necrotic tissue in relation to the whole LV (viable myocardium plus necrosis).

### Statistics

Values were tested for a Gaussian distribution using the D’Agostino–Pearson omnibus normality test with a 95% confidence level or Kolmogorov–Smirnov test. Continuous variables are presented as medians ± lower and upper quartiles if they followed a non-Gaussian distribution and as means ± SEM if they followed a Gaussian distribution. Non-normally distributed variables were tested using the Mann–Whitney U-test for unpaired analysis and the Wilcoxon matched-pairs side rank test for paired analysis. Normally distributed values were tested using unpaired or paired Student’s T-test. Differences between more than two groups were compared using Kruskal–Wallis test or ordinary one-way ANOVA, respectively. Sample size calculation for the comparative analysis was estimated from a previous study using echocardiography data from C57BL/6 male for 95% power at the 0.05 level of significance to detect a difference in Ejection Fractions. All figures are presented as mean ± SEM. In all cases, a *p* < 0.05 was considered statistically significant.

### Supplementary Information

Supplementary Figures.

## Data Availability

The data that support the findings of this study are available from the corresponding author upon reasonable request.

## Abbreviations

NLRP3: NLR family pyrin domain containing 3
IL-1β: Interleukin-1β
MI: Myocardial infarction
ASC: Apoptosis-associated speck-like protein containing a CARD
EF: Ejection fraction
EDV: End diastolic volume
ESV: End systolic volume
FC: Flow cytometry
FS: Fractional shortening
GSDMD: Gasdermin D
HF: Heart failure
ICAM-1: Intercellular adhesion molecule-1
LAD: Left anterior descending artery
LV: Left ventricle
NET: Neutrophil extracellular trap
PAD4: Protein arginine deiminase 4
PECAM-1: Platelet endothelial cell adhesion molecule 1
TF: Tissue factor
TNF-α: Tumor necrosis factor alpha
TTC: Triphenyle tetrazolium chloride staining
VCAM-1: Vascular cell adhesion molecule-1
VWF: Von Willebrand factor
WT: Wild type
