# Supplementary figures and images for: Neutrophil NLRP3 promotes cardiac injury following acute myocardial infarction through IL-1β production, VWF release and NET deposition in the myocardium
Source: Sci Rep. 2024 Jun 24;14:14524. doi: 10.1038/s41598-024-64710-4 (PMC11196583; doi:10.1038/s41598-024-64710-4)

Supplement Figure 1

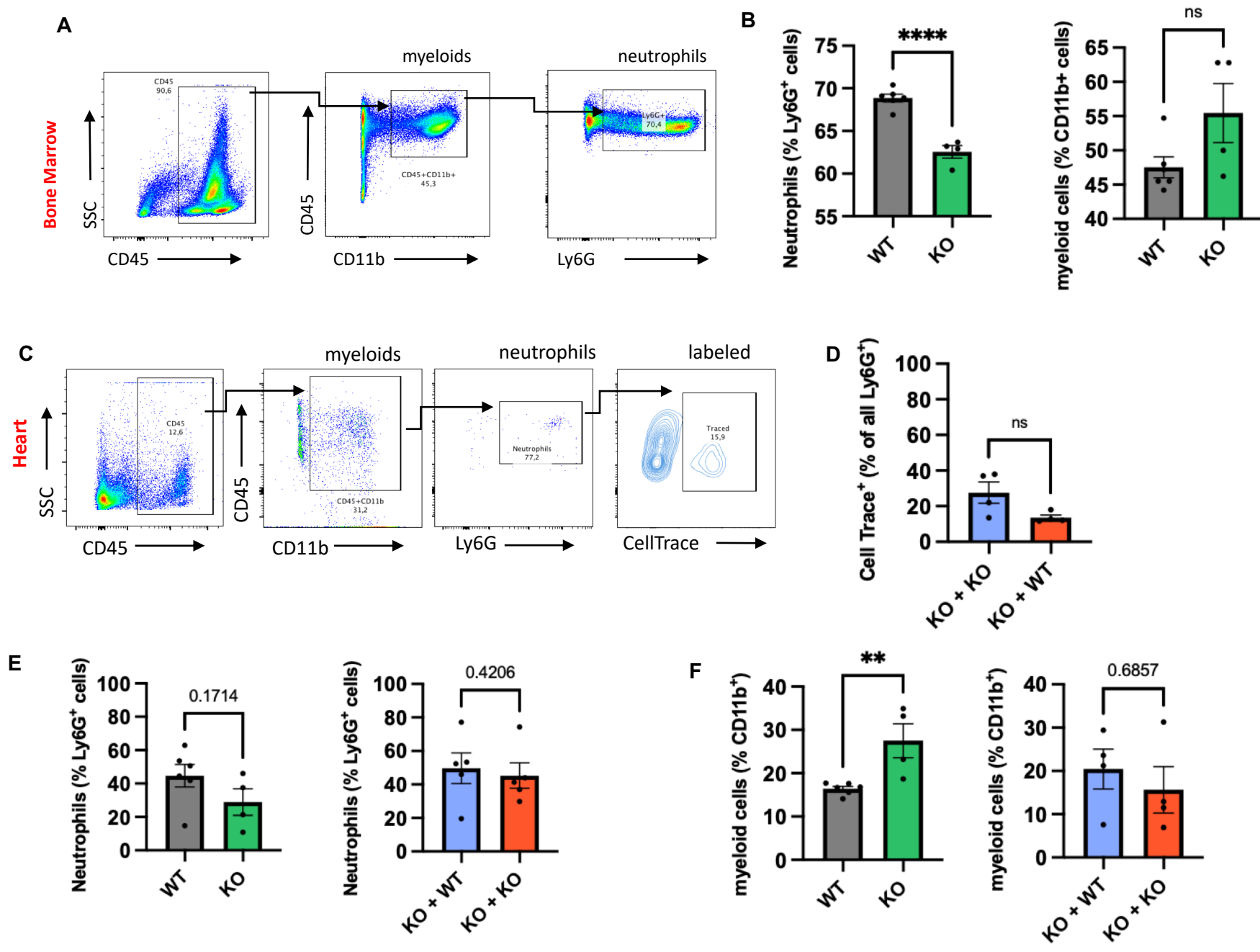

Supplement Figure 2

A

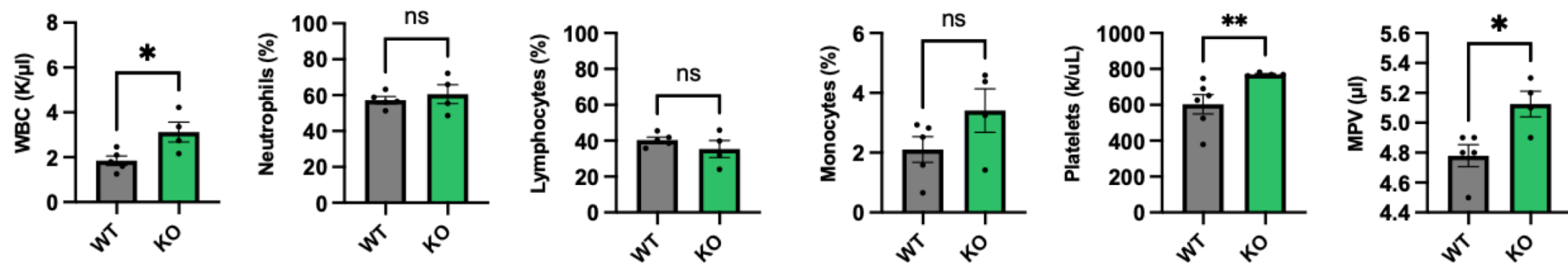

B

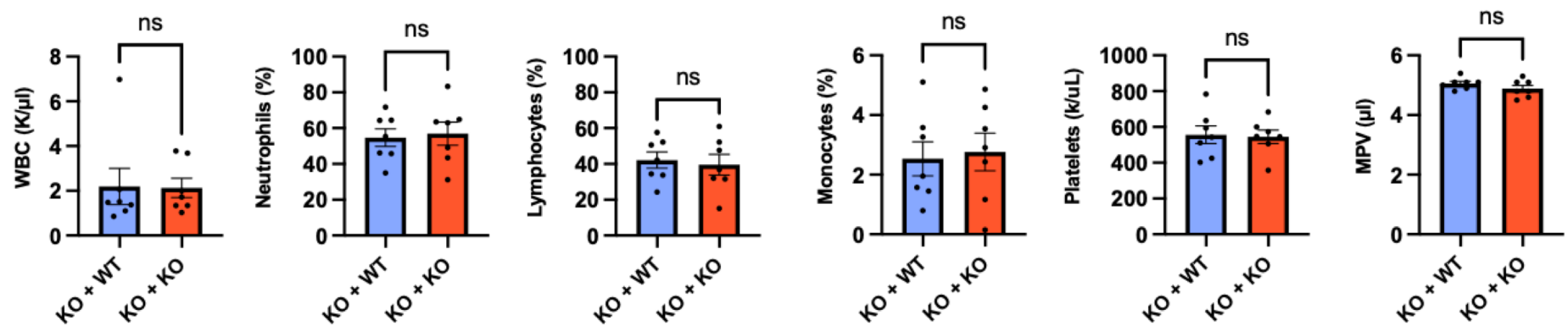

Supplement Figure 3

A

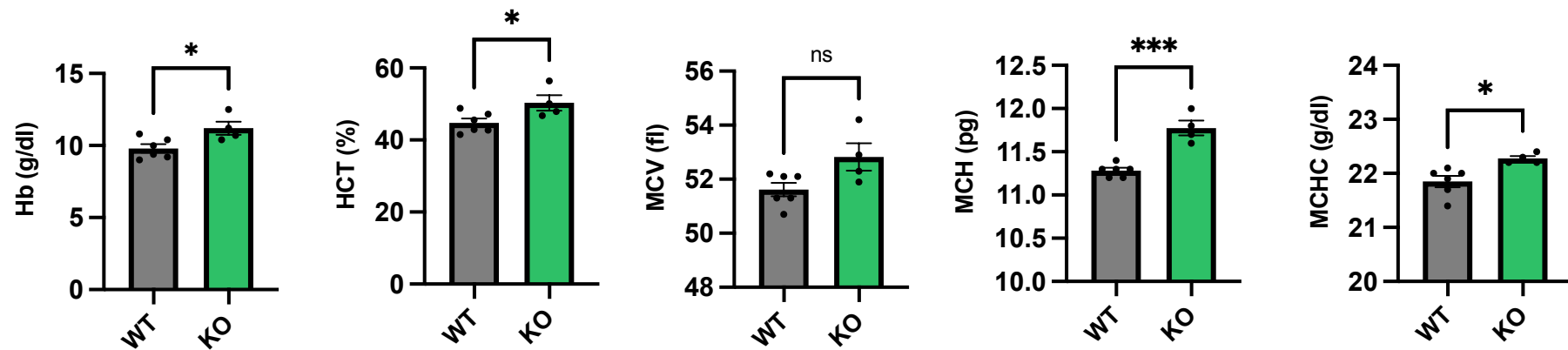

B

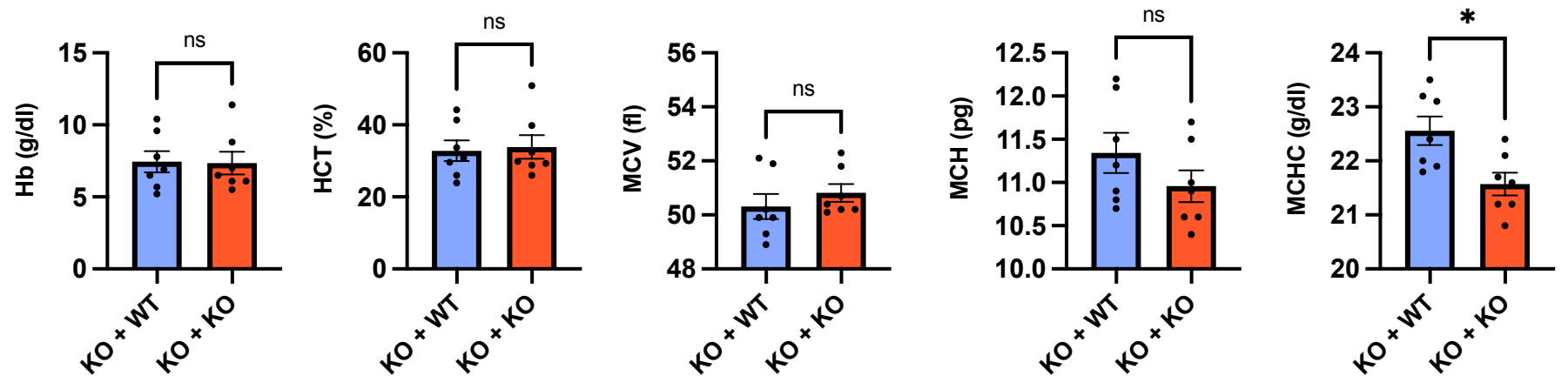

Supplement: Supplementary file 1 — Supplementary Figures. [file 41598_2024_64710_MOESM1_ESM.pdf]
